# Monitoring SARS-CoV-2 variants with complementary surveillance systems: risk evaluation of the Omicron JN.1 variant in France, August 2023 to January 2024

**DOI:** 10.2807/1560-7917.ES.2025.30.1.2400293

**Published:** 2025-01-09

**Authors:** Adriana Traore, Kelly Charniga, Sophie Grellet, Garance Terpant, Héléna Da Cruz, Anais Lamy, Nathalie Thomas, Gwladys Gbaguidi, Alizé Mercier, Julie Prudhomme, Benoit Visseaux, Vincent Vieillefond, Stéphanie Haim-Boukobza, Jean-Marc Giannoli, RELAB Study Group, Laboratory group, Javier Castro-Alvarez, Alain-Claude Kouamen, Marie-Anne Rameix-Welti, Samar Beirrera-Ibraim, Gregory Destras, Laurence Josset, Simon Cauchemez, Bruno Lina, Bruno Coignard, Justine Schaeffer, Vincent Enouf, Antonin Bal, Members of the RELAB Study Group, Arnaud François, Alexandre Vignola, Vincent Garcia, Alexandra Jacques, Jonas Amzalag, Nadège Gourgouillon, Remi Labetoulle, Frederique Roumanet, Arthur Denoel, Hilel Mehamha, Thierry Guffond, Magali Hypolite, Yanis Chaib, Timothée Goetschy, Elodie Ostermann, Anne Holstein, Jean Marc Giannoli, Julienne de Pontcharra, Jean Francois Comes, Justine Gasnot, Theo Corbet, Laurent Kbaier, Emmanuel Chanard, Arcadie Gioud, Stéphanie Arsene, Maxime Sansot, Anne-Lise Gautier, Kariach Goldar, Mahery Ramiandrisoa, Aristide Nzeumi, Pauline Jestin, Gilles Abs, Guillemette Wandler, Anne-Claire Strzelecki, Sarah Cerdan, Edouard Delaunay, Sandrine Barrieu-Moussat, Laurence Prots, Edouard Delaunay, Johanna Roux, Yasmina De Saint Salvy, Agnes Durand, Aude Lesenne, Kader Merah, Erwan Le Naour, David Robert, Sophie Zaffreya, Jean-Philippe Galhaud, Claire Felloni, Dominique Dyda, Aurelie Dupuis, Gwenole Prigent, Stephanie Arsene, Antoine Prigent, Natacha Tatoyan, Bénédicte Roquebert, Odile Rousselet, Michel Sala, Members of the Laboratory Group, Slim Fourati, Astrid Vabret, Meriadeg Legouil, Celine Boschi, Bernard La Scola, Stephanie Gouarin, Agathe Boudet, Sonia Burrel, Veronique Avettand-Fenoel, Amal Chaghouri, Georges Dos-Santos, Ilka Engleman, Floriane Gallais, Alexandre Gaymard, Jerome Le-Goff, Quentin Lehingrat, Nicolas Leveque, Stéphane Sylvain Marot, Audrey Mirand, Lina Mouna, Jean-Michel Pawlotsky, Sylvie Pillet, Jean-Christophe Plantier, Charlotte Pronier, Sylvie Rogez, Maud Salmona, Véronique Brodard, Aurélie Schnuriger, Cécile Henquell, Nael Zemali, Alice Moisan, Richard Njouom, Marie-Christine Jaffar-Bandjee, Laurent Souply, Nefert Dossou, Theophile Cocherie, Fairly Warnakulasuriya, Enagnonkazali Alidjinou, Elise Bouthry, Segolene Brichler, Lionel Chollet, Anne Demonte, Julia Dina, Antoine Enfissi, Samira Fafi-Kremer, Catherine François, Elyanne Gault, Linda Handala, Berthemarie Imbert, Jacques Izopet, Caroline Lefeuvre, Soizic Lemestre, Stéphanie Marque Juillet, Audrey Merens, Cecile Moins, Lea Pilorge, Cecile Poggi, Alexandre Regueme, Christophe Rodriguez, Dominique Rousset, Sandrine Castelain, Etienne Simon-Loriere, Morgan Solis, Cathia Soulie, Anne-Lise Toyer, Pauline Tremeaux, Sophie Vallet, Philippe Colson

**Affiliations:** 1 Direction des Maladies Infectieuses, Santé publique France, Saint-Maurice, France; 2 Mathematical Modelling of Infectious Diseases Unit, Institut Pasteur, Université Paris Cité, CNRS UMR 2000, Paris, France; 3 Direction des Régions, Santé publique France, Saint-Maurice, France; 4 Cerba, Infectiology Department, Saint-Ouen-l’Aumône, France; 5 BPO-BIOEPINE-Biogroup, Levallois-Perret, France; 6 Laboratoires Cerballiance, Issy-Les-Moulineaux, France; 7 LABAC, Villeurbanne, France; 8 The members of RELAB Study Group are listed under Collaborators and at the end of the article; 9 The members of the Laboratory Group are listed under Collaborators; 10 National Reference Center for Respiratory Viruses, M3P, Institut Pasteur, Université Paris Cité, Paris, France; 11 M3P, Institut Pasteur, Université Paris-Saclay, Université de Versailles St. Quentin, Université Paris Cité, UMR 1173 (2I), INSERM; Assistance Publique des Hôpitaux de Paris, Hôpital Ambroise Paré, Paris, France; 12 National Reference Center for Respiratory Viruses, Hospices Civils de Lyon, CIRI, INSERM U1111, University Claude Bernard Lyon 1, Lyon, France; * These authors have contributed equally to this work.

**Keywords:** COVID-19, genomic surveillance, SARS-CoV-2 variant, JN.1, BA.2.86, laboratory network

## Abstract

**Background:**

Early detection and characterisation of SARS-CoV-2 variants have been and continue to be essential for assessing their public health impact. In August 2023, Santé publique France implemented enhanced surveillance for BA.2.86 and sub-lineage JN.1 because of their genetic divergence from other variants and increased prevalence.

**Aim:**

To detail how combining epidemiological and laboratory data sources, targeted investigations and modelling enabled comprehensive characterisation of sub-lineage JN.1.

**Methods:**

Data were collected from epidemiological investigations using a standardised questionnaire and from routine and novel (RELAB network) surveillance systems. JN.1 cases were compared with cases infected with previously circulating variants, such as EG.5, BA.4/BA.5 and other BA.2.86 sub-lineages. The growth rate and doubling time of JN.1 were estimated.

**Results:**

JN.1 was first detected in September 2023 in the Île-de-France region, France, and spread widely across the country. By late November, doubling time was estimated to be 8.6 to 26.4 days depending on the region. For all data sources, cases infected by JN.1 showed similar demographics, rates of hospitalisation and RT-PCR cycle threshold values compared with those infected by previous variants. JN.1 cases also had older median age (54 years; 40–71 vs 47 years; 30–59), more frequent reports of feverish feeling and less frequent cough or nausea compared with BA.4/BA.5 cases. JN.1 cases had significantly higher frequency of anosmia compared with other BA.2.86 cases.

**Conclusion:**

Combining different data sources played a key role in detecting emerging variant JN.1, for which no evidence of increased public health impact was found despite its genetic divergence.

**Key public health message**

**What did you want to address in this study and why?**

SARS-CoV-2, the virus causing COVID-19, is constantly evolving. It remains essential to monitor SARS-CoV-2 variants and evaluate their potential impact. In August 2023, a new variant named BA.2.86 raised concerns in France, as it was genetically very different from other variants circulating at the time. We reinforced epidemiological and clinical surveillance of BA.2.86 and its sub-lineage JN.1 to evaluate the need for dedicated control measures.

**What have we learnt from this study?**

The diversity of data sources from our routine surveillance system allowed us to detect and monitor emergence of BA.2.86 and JN.1 early and to obtain accurate and useful data. We collaborated with a modelling team to study the spread of these new variants in France and initiated targeted investigations of BA.2.86 cases, in collaboration with a new network of community-based laboratories RELAB to gather more information on a public health signal.

**What are the implications of your findings for public health?**

This study found no evidence of increased public health impact of JN.1 compared with previously circulating variants, despite its genetic differences. In addition, we showed the benefit of combining various surveillance systems and complementary investigations that can be triggered on-demand. The data obtained from the novel network of community-based laboratories (RELAB network) proved to be useful for respiratory virus surveillance.

## Introduction

During the COVID-19 pandemic, one of the challenges for the public health response was the early detection of emerging SARS-CoV-2 variants and the rapid evaluation of their potential public health impact. France adapted existing surveillance systems, such as the syndromic surveillance system (SurSaUD) and a crisis information system centralising data on hospitalisations (SI-VIC), and developed new systems, such as an exhaustive and central repository for SARS-CoV-2 testing results (SIDEP) and wastewater surveillance (SUM’EAU). As variants of SARS-CoV-2 began to emerge in late 2020, they changed the dynamics of the pandemic and triggered the (re-)introduction of control measures. To respond to these challenges, the EMERGEN consortium (Consortium for surveillance and research on EMERging pathogens using GENomics), piloted by the French national public health agency Santé publique France (SpF) and the National Agency for Research on AIDS and Emerging Infectious Diseases – National Institute of Health and Medical Research (ANRS-MIE – Inserm), was created in early 2021 [1]. This consortium brought together French actors of surveillance and research on emerging infectious diseases to support the efforts for SARS-CoV-2 sequencing.

In 2023, the landscape of SARS-CoV-2 variants was dominated by the Omicron XBB recombinant and its sub-lineage, EG.5. By mid-August, a new variant, later designated as BA.2.86, was detected in Denmark and Israel [2]. Compared with its closest parental lineage BA.2, BA.2.86 exhibited over 30 mutations in its spike protein, including mutations previously associated with immune escape. This genetic divergence and its subsequent spread to multiple countries led to the classification of BA.2.86 as variant of interest (VOI) by the World Health Organization (WHO) on 21 November 2023 [3].

In France, BA.2.86 was first detected on 31 August 2023 in the Grand Est region before spreading to other regions. As soon as BA.2.86 was detected, SpF initiated enhanced surveillance of BA.2.86 to assess its potential impact. To complement data from passive surveillance, BA.2.86 cases that were confirmed through sequencing by the National Reference Center for Respiratory Viruses (NRC-VIR) or laboratories from the EMERGEN consortium were investigated [1].

**Figure 1:**
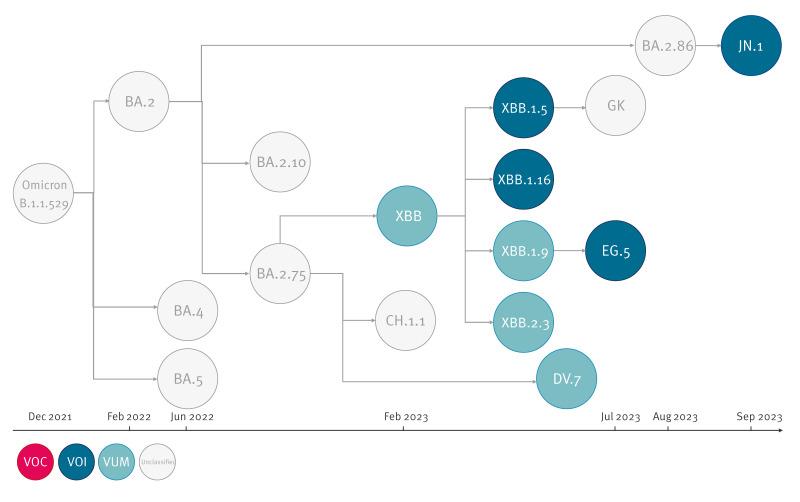
Schematic illustration of the genetic lineage of SARS-CoV-2 Omicron variants, December 2021–September 2023

VOC: variant of concern; VOI: variant of interest; VUM: variant under monitoring.

Genetic diversification within BA.2.86 led to the emergence of sub-lineages, including JN.1, which was classified as a VOI by WHO on 18 December 2023 [4]. Compared with its parental lineage BA.2.86.1, JN.1 only carries three amino acid substitutions, including one in its spike protein (L455S). France was one of the first countries to identify JN.1 and report a rapid rise in cases [5]. A schematic illustration of classified and unclassified Omicron variants circulating in France is shown in Figure 1.

The nomenclature follows the classification of the World Health Organization [26].

In this study, we aimed to detail how combining epidemiological, sequencing and laboratory data sources, targeted investigations and modelling enabled comprehensive characterisation of this novel SARS-CoV-2 variant JN.1 and provide an assessment of its potential public health impact in France.

## Methods

### Data sources

**Figure 2:**
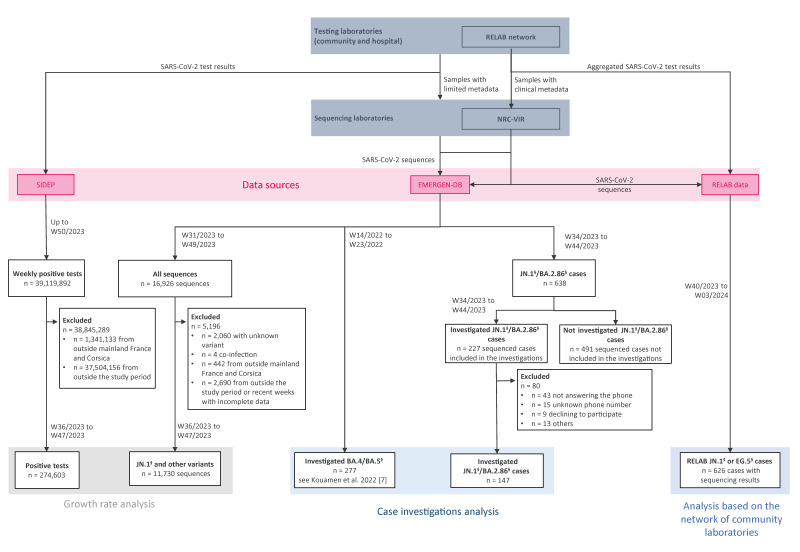
Flowchart of the generation of each dataset used in this study, France, February 2024

For this study, we used data collected through several surveillance systems to monitor COVID-19 in France. The data sources are described below, and their inter-connection is illustrated in Figure 2.

NRC-VIR: National Reference Center for Respiratory Viruses.

^§^ Indicates that all sub-lineages of a variant are included.

This flowchart includes time periods considered, exclusion criteria, number of included cases and interconnection between the data sources. The three data sources are detailed in the Methods.

SIDEP [6] is an exhaustive and central repository for SARS-CoV-2 testing results. It collects test results in real time from SARS-CoV-2 reverse transcriptase-PCR (RT-PCR) and lateral flow tests performed by hospitals and community-based laboratories across France. Data are pseudonymised and include information about the patient (sex, age, city of residence, whether they are a healthcare professional, symptom onset), the test (sampling date, sampling laboratory) and the result (result date, type of analysis, test result) in line list format.

The EMERGEN database (EMERGEN-DB) [1] is a genomic surveillance database that collects SARS-CoV-2 sequences (raw and assemblies) and associated metadata from sequencing laboratories of the EMERGEN consortium. Co-piloted by SpF and ANRS-MIE, the surveillance relied on a network of both public and private sequencing laboratories. The database was previously hosted at the French Institute of Bioinformatics (IFB) and migrated the end of June 2024 to the Mixed Service Unit 56 (UMS56) of the University Hospital Institute (IHU Aix-Marseille - Inserm). All sequencing results produced by the consortium are submitted to national (EMERGEN-DB) and international (GISAID) databases. Sequences uploaded to EMERGEN-DB are regularly reanalysed to account for changes in the Nextstrain (clade) and Pango (lineage) nomenclatures [7,8]. In this publication, the section sign (§) indicates that all sub-lineages of a variant are included. ‘Other BA.2.86^§^’ refers to BA.2.86 sub-lineages excluding JN.1 and its sub-lineages.

RELAB is a novel network of community-based laboratories established in September 2023 [9]. It is coordinated by the NRC-VIR and includes two large networks of private laboratories, BIOGROUP and CERBALLIANCE. A total of 1,600 laboratories send clinical and virological data from their respiratory virus testing by RT-PCR (SARS-CoV-2, influenza virus, respiratory syncytial virus triplex RT-PCR) to the NRC-VIR on a weekly basis, as well as samples for further characterisation including viral isolation and whole genome sequencing (ARTIC amplicons and Illumina sequencing) as previously described [10]. We analysed RELAB data between 2 October 2023 (week 40) and 18 January 2024 (week 3), when JN.1^§^ and EG.5^§^ co-circulated.

### Case investigations

Until week 44 of 2023, laboratories from the NRC-VIR and the EMERGEN consortium reported all BA.2.86^§^ cases identified to SpF and uploaded the sequences to EMERGEN-DB (Figure 2). Because of sequencing delays, sampling dates from these cases ranged from 21 August 2023 (week 34) to 5 November 2023 (week 44). Epidemiologists from SpF regional offices contacted all of these cases for an interview. Using a standardised questionnaire, demographic (sex, age, region), exposure (travel history abroad or to overseas territories within 14 days before symptom onset), cluster (report of known cases among contacts), clinical symptoms (date of symptom onset or absence of symptoms; asthenia/fatigue, fever, headache, runny nose, cough, myalgia, sore throat, feverish feeling, anosmia, shortness of breath, ageusia, diarrhoea, nausea/vomiting, abnormal lung auscultation, acute respiratory distress syndrome or other), outcome (hospitalisation and intensive care admissions, dates of admission and discharge, and death), pre-existing conditions (hypertension, obesity, diabetes, chronic respiratory disease, renal insufficiency, cancer, immunosuppression, liver disease, heart disease, neuromuscular pathology, pregnancy or other), previous SARS-CoV-2 infection and vaccination status (number of doses and date of administration) were collected [11].

The same methodology and the same questionnaire had been previously used to investigate BA.4/BA.5^§^ cases when these variants emerged in spring 2022. BA.4/BA.5^§^ cases were investigated between 6 April 2022 (week 14) and 10 June 2022 (week 23), and the results of these investigations are available in Kouamen et al. [7]. These cases were used in the study as a comparison group.

### Descriptive analysis

The representativeness of investigated cases was assessed by comparing sex, age, region and date of diagnosis between investigated cases and all sequenced BA.2.86^§^ cases from EMERGEN-DB over the study period (data exported on 11 December 2023 (week 50)). JN.1^§^ cases were also compared with BA.4/BA.5^§^ cases from previous investigations.

Using RELAB data, demographic (age and sex), clinical (fever, respiratory symptoms, date of symptom onset), vaccination and virological (semi-quantitative viral load assessment with cycle threshold values, Ct) data were compared between JN.1^§^ and EG.5^§^ cases.

The statistical analyses and graphs were produced using R (The R Foundation for Statistical Computing). Wilcoxon rank sum test was used for continuous variables and Chi-square or Fisher’s exact test for categorical variables. Differences were considered to be statistically significant when the p value was below 0.05.

### Growth rate/doubling time of JN.1^§^ cases

#### Time period and area under study

For the analysis of growth rate/doubling time of the JN.1 variant, the time period of interest was epidemiological weeks 31–49 of 2023 (the time corresponding to the availability of the EMERGEN-DB data when the analysis was performed). The geographic area under study included mainland France (mainland France designates the French territory without its overseas territories, see the map in Supplementary Figure S1).

#### Data sources

Two data sources were used for the modelling component of this analysis: (i) the number of SARS-CoV-2 samples with variant typing results from EMERGEN-DB and (ii) the weekly number of positive tests for SARS-CoV-2 (COVID-19 cases) from SIDEP.

#### Data cleaning

A flowchart containing exclusion criteria of observations for both datasets can be found in Figure 2.

For EMERGEN-DB data, 16,926 sequences were available between 31 July 2023 (week 31) and 10 December 2023 (week 49). Of these, 2,060 sequences were removed because the variant was unknown, along with four samples that were co-infections and 442 sequences from outside mainland France and Corsica. Overseas regions and departments were removed because SARS-CoV-2 transmission dynamics were likely different in those places. Data from Corsica were excluded because of limited sample size that would likely have biased the analyses. As data appeared incomplete for the most recent 2 weeks (weeks 48 and 49) and because the JN.1 variant was not detected in France until early September, the time series was truncated to weeks 36–47.

For SIDEP data, there were 39,119,892 positive tests for SARS-CoV-2 (COVID-19 cases) available from 11 May 2020–11 December 2023 (weeks 20–50). Positive tests from outside mainland France and Corsica were removed (n = 1,341,133). We also removed 37,504,156 positive tests from weeks outside the updated study period (weeks 36–47, to match the EMERGEN-DB data).

#### Estimating the number of COVID-19 cases by variant

For each region, the proportion of samples that tested positive for SARS-CoV-2 was calculated for (i) JN.1^§^ and (ii) all other variants. Then these proportions were multiplied by the number of positive tests for SARS-CoV-2 by region from the SIDEP data and plotted. To obtain the weekly number of estimated COVID-19 cases nationally, the estimated number of cases across regions were summed.

#### Estimating growth rates and doubling times of JN.1^§^

Log-linear models were fitted to the weekly estimated number of COVID-19 cases caused by JN.1^§^ for each region using the incidence R package [8]. Parameter estimates were extracted for growth rates, doubling times and incidence predictions along with their 95% confidence intervals.

## Results

### Emergence of JN.1^§^ in France

**Figure 3:**
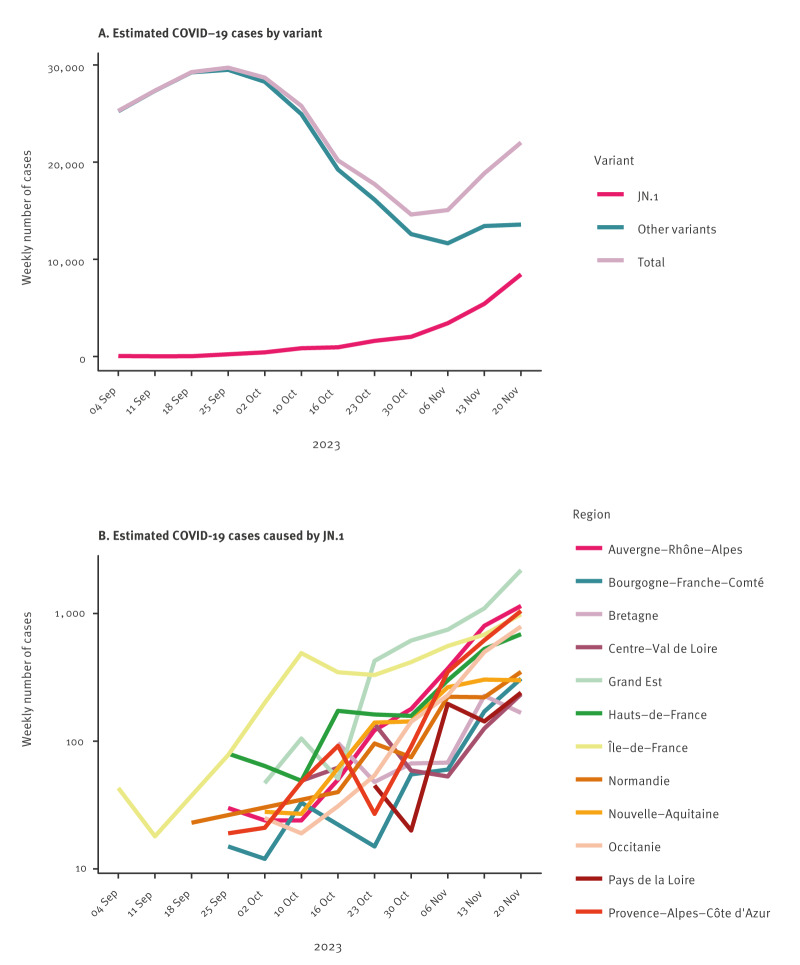
Weekly estimated number of COVID-19 cases for mainland France^a^, weeks 36–47 2023

Until October 2023, the number of COVID-19 cases in France was decreasing. However, cases started to rise again in November and December. This rise was associated with the spread of the JN.1^§^ variant, which was first detected in the Île-de-France region in September 2023 (Figure 3).

SARS-CoV-2: severe acute respiratory syndrome coronavirus 2.

^a^ Mainland France excluding Corsica.

Estimated cases are coloured by region and plotted on a log10 scale, from weeks 36–47 of 2023. ‘Other variants’ correspond to non-JN.1^§^ SARS-CoV-2 samples.

For the analysis of growth rates/doubling times, there were 11,730 sequences remaining for analysis from EMERGEN-DB. For SIDEP, for a total of 274,603 positive tests were analysed for weeks 36–47 of 2023. The growth rate of JN.1^§^ cases varied between regions from 0.081 (Occitanie region) to 0.026 (Centre-Val de Loire region) by the end of November. Doubling time ranged from 8.6 days (Occitanie region) to 26.4 days (Centre-Val de Loire region, Table 1). The doubling time for three regions (Bretagne, Centre-Val de Loire and Pays de la Loire) was highly uncertain with undefined 95% confidence intervals. Incidence predictions and 95% confidence intervals from the log-linear models are shown in Supplementary Figure S2.

**Table 1:** Growth rates and doubling times (days) of JN.1^§^ by region, France, weeks 36–47 2023

Region	Growth rate	95% CI	Doubling time in days	95% CI
Auvergne-Rhône-Alpes	0.076	0.059 to 0.093	9.1	7.4 to11.8
Bourgogne-Franche-Comté	0.052	0.026 to 0.079	13.3	8.8 to 27.1
Bretagne	0.030	−0.017 to 0.077	23.0	undefined
Centre-Val de Loire	0.026	−0.008 to 0.060	26.4	undefined
Grand Est	0.080	0.052 to 0.108	8.7	6.4 to 13.2
Hauts-de-France	0.044	0.028 to 0.060	15.8	11.5 to 24.8
Île-de-France	0.046	0.031 to 0.061	15.2	11.4 to 22.7
Normandie	0.045	0.027 to 0.062	15.4	11.1 to 25.3
Nouvelle-Aquitaine	0.056	0.036 to 0.077	12.3	9.1 to 19.2
Occitanie	0.081	0.063 to 0.099	8.6	7.0 to 11.1
Pays de la Loire	0.076	−0.032 to 0.184	9.1	undefined
Provence-Alpes-Côte d'Azur	0.072	0.045 to 0.099	9.7	7.0–15.5

CI: confidence interval.

Growth rate has no units. Analysis period: 4 September to 20 November 2023.

Doubling times for Bretagne, Centre-Val de Loire, and Pays de la Loire were highly uncertain. The 95% CI for the growth rate overlapped with 0, and because doubling time is defined as ln(2)/growth rate, the confidence intervals for doubling time were undefined.

### Comparison between JN.1^§^, other BA.2.86^§^ and BA.4/BA.5^§^ cases

Among the 227 cases of BA.2.86^§^ reported between 21 August 2023 (week 34) and 5 November 2023 (week 44), 147 cases (65%) were investigated (see Supplementary Figure S3 for time distribution of BA.2.86 sequences (investigated cases or cases not investigated)). Eighty cases (37%) could not be investigated because of not answering the phone (54%, n = 43), unknown phone number (19%, n = 15), deceased not linked to COVID-19 or partial data (16%, n = 13) or declining to participate (11%, n = 9). The investigated cases had similar sociodemographic characteristics compared with all BA.2.86^§^ cases available in the EMERGEN-DB. More details on variant, Pango lineage, sex, age and region of residence are provided in Supplementary Table S1. The investigated cases represented 23% of all BA.2.86^§^ cases available in the EMERGEN-DB (n = 638), with half of them corresponding to JN.1^§^.

### Comparison of JN.1^§^ cases vs other BA.2.86^§^ cases

Among the 147 investigated cases, 74 belonged to JN.1^§^ and 73 to other BA.2.86^§^. Investigated JN.1^§^ cases resided in 12 of the 18 French regions (with one of the overseas territories). The median age of the JN.1^§^ cases was 54 years (interquartile range (IQR): 40–71). The sex ratio was 0.6 females to males for both JN.1^§^ and BA.2.86^§^ (Table 2).

**Table 2:** Characteristics of investigated JN.1^§^ cases (n = 74) compared to other BA.2.86^§^ (n = 73) and BA.4/BA.5^§^ (n = 277) cases, France, 2022–2023

Characteristics	Investigated cases from weeks 34–44/2023					Investigated cases from weeks 14–23/2022		
	JN.1^§^ (n = 74)^a^		Other BA.2.86^§^ (n = 73)^a^		p value^b^	BA.4/BA.5^§^ (n = 277)^a^		p value^b^
	n	%	n	%		n	%	
**Sex**								
Total	74	100	73	100	0.57	275	100	0.44
Female	47	63.5	43	58.9		161	58.5	
Male	27	36.5	30	41.1		114	41.5	
**Age**								
Total	74	100	72	100	0.22	268	100	0.003
Median (IQR)	53.5 (40.0–70.8)		61.5 (42.8–72.0)			47.4 (30.2–59.4)		
**Symptoms**								
Total	72	100	73	100	1	275	100	1
Asymptomatic	2	2.8	2	2.7		9	3.3	
Symptomatic	70	97.2	71	97.3		266	96.7	
**Duration of symptoms in days**								
Total	63	100	59	100	0.28	244	100	0.94
Median (IQR)	7.0 (4.0–8.0)		7.0 (4.0–11.0)			7.0	(4.0–10.2)	
**Hospitalisation**								
Total	69	100	68	100	**0.02**	266	100	1
Yes	3	4.3	13	18.8		12	4.5	
No	66	95.7	56	81.2		254	85.5	
**Intensive care **								
Total	69	100	68	100	0.50	266	100	1
Yes	0	0	1	1.4		0	0	
No	69	100	68	98.6		266	100	
**Death**								
Total	69	100	68	100	1	266	100	1
Yes	0	0	0	0		0	0	
No	69	100	68	100		266	100	
**Previous SARS-CoV-2 infection(s)**								
Total	68	100	67	100	0.26	266	100	**< 0.001**
Yes	42	61.8	35	52.2		38	14.3	
No	26	38.2	32	47.8		228	85.7	
**Risk factors **								
Total	70	100	71	100	0.80	271	100	** 0.005**
Yes	33	47.1	35	49.3		78	28.8	
No	37	52.9	36	50.7		193	71.2	
**Vaccination status** ^c^								
Total	68	100	67	100	0.27	271	100	**< 0.001 **
Unvaccinated	14	20.6	10	14.9		55	20.3	
One dose	3	4.4	0	0		1	0.4	
Two doses	8	11.8	7	10.4		39	14.4	
Three doses	27	39.7	33	49.3		174	64.2	
More three doses	16	23.5	17	25.4		2	0.7	
**Travel **								
Total	69	100	68	100	0.16	275	100	0.06
Yes	5	7.2	10	14.7		44	16.0	
No	64	92.8	58	85.3		231	84.0	
**COVID-19 cluster**								
Total	69	100	63	100	**0.009**	268	100	0.33
Yes	30	43.5	14	22.2		134	50.0	
No	39	56.5	49	77.8		134	50.0	
**Region of residence **								
Total	74	100	73	100	0.34	277	100	**< 0.001**
Auvergne-Rhône-Alpes	4	5.4	8	11.0		27	9.7	
Bourgogne-Franche-Comté	5	6.8	10	13.7		26	9.4	
Bretagne	2	2.7	2	2.7		61	22.0	
Centre-Val de Loire	7	9.5	9	12.3		14	5.1	
Corsica	0	0	3	4.1		7	2.5	
Grand Est	6	8.1	6	8.2		15	5.4	
Guadeloupe	0	0	0	0		0	0	
Guyane	0	0	0	0		1	0.4	
Hauts-de-France	9	12.2	4	5.5		21	7.6	
Île-de-France	23	31.1	14	19.2		16	5.8	
La Réunion	7	9.5	5	6.8		3	1.1	
Martinique	0	0	0	0		17	6.1	
Mayotte	0	0	0	0		0	0	
Normandie	1	1.4	0	0		13	4.7	
Nouvelle-Aquitaine	0	0	2	2.7		24	8.7	
Occitanie	3	4.1	5	6.8		19	6.9	
Pays de la Loire	1	1.4	1	1.4		2	0.7	
Provence-Alpes-Côte d'Azur	6	8.1	4	5.5		11	4.0	

IQR: interquartile range; SARS-CoV-2: severe acute respiratory syndrome coronavirus 2.

^a^ For each category, percentages have been calculated for the number of cases with available data.

^b^ Wilcoxon rank sum test was used for continuous variables. Chi-square test was used for categorical variables with Fisher's exact test for small theoretical numbers. Bold indicates statistical significance at the 0.05 level.

^c^ Vaccines that have been granted marketing authorisation in France, as outlined in [25].

Less than 5% of JN.1^§^ cases were hospitalised following infection, which is less than that for other BA.2.86^§^ cases. None of the investigated cases were admitted into intensive care units and no deaths linked to COVID-19 were reported (Table 2).

**Figure 4:**
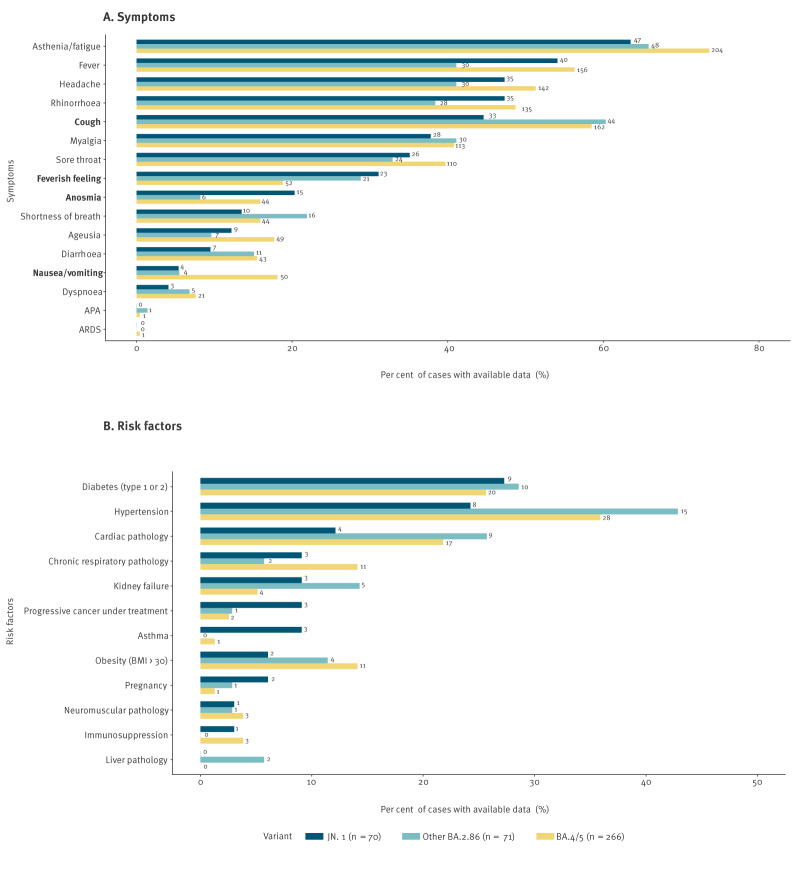
Proportion of symptoms (A) and risk factors (B) reported by JN.1^§^ cases (n = 70) compared with previously investigated other BA.2.86^§^ cases (n = 71), and BA.4/BA.5^§^ (n = 266) cases, France, 2022–2023

APA: abnormal pulmonary auscultation; ARDS: acute respiratory distress syndrome; BMI: body mass index.

Most JN.1^§^ cases developed symptoms (97.2%). The most common symptoms were asthenia/fatigue (67.1%), fever (57.1%), headache (50.0%), rhinorrhoea (50.0%) and cough (47.1%) (Figure 4A). Anosmia was significantly more frequent among JN.1^§^ cases than other BA.2.86^§^ cases (21.4% vs 8.5%, p = 0.03). Further details on the proportion of symptoms of JN.1^§^ cases compared with to previously investigated cases are provided in Supplementary Table S2. Median duration of symptoms was 7 days (IQR: 4–10) across all variants considered (Figure 4A). 

Absolute numbers of cases are shown beside the bars. The bars are in descending order according to the most common symptoms/risk factors. Four symptoms are statistically significant and marked with bold text. Anosmia was significantly more frequent among JN.1^§^ cases than other BA.2.86^§^ cases (21.4% vs 8.5%, p = 0.03). Cough was significantly less frequent among JN.1^§^ cases than other BA.4/BA.5^§^ cases (47.1% vs 58.5%, p = 0.03). Feverish feeling was significantly more frequent among JN.1^§^ cases than other BA.4/BA.5^§^ cases (32.9% vs 18.8%, p = 0.02). Nausea/vomiting was significantly less frequent among JN.1^§^ cases than other BA.4/BA.5^§^ cases (5.7% vs 18.1%, p = 0.007).

Nearly half of JN.1^§^ cases (47.1%) had at least one risk factor for developing a severe form of COVID-19, which was not significantly different compared with other BA.2.86^§^ cases. The most frequent risk factors were diabetes (27.3%) and high blood pressure (24.2%) (Figure 4B).

Most JN.1^§^ cases (92.8%, comparable to other BA.2.86^§^) did not mention having travelled abroad and nearly half of JN.1^§^ cases (43.5%) were linked to a COVID-19 cluster, which was higher than for other BA.2.86^§^ cases (22.2%, Table 2).

At the time of investigation, more than half of JN.1^§^ cases (61.8%) reported a previous SARS-CoV-2 infection (comparable to other BA.2.86^§^). Regarding vaccination status, no statistically significant difference was found compared with other BA.2.86^§^ cases, with 20.6% of JN.1^§^ investigated cases being unvaccinated, 4.4% vaccinated with one dose, 11.8% with two doses, 39.7% with three doses and 23.5% with more than three doses of COVID-19 vaccine (Table 2).

### Comparison of JN.1^§^ characteristics with BA.4/BA.5^§^

There was no significant difference in the number of hospitalised cases or the number of cases who reported travel history between JN.1^§^ and BA.4/BA.5^§^ cases. The median age was 47 years (IQR: 30–59) for BA.4/BA.5^§^ cases (Table 2).

JN.1^§^ cases more frequently declared feverish feeling than BA.4/BA.5^§^ cases (32.9% vs 18.8%, p = 0.02) but less frequently declared cough symptoms (47.1% of JN.1^§^ cases vs 58.5% of BA.4/BA.5^§^ cases, p = 0.03) or nausea/vomiting (5.7% of JN.1^§^ cases vs 18.1% of BA.4/BA.5^§^ cases, p = 0.007; Figure 4A. Additional details on the proportion of symptoms of JN.1^§^ cases compared with previously investigated cases are provided in Supplementary Table S2.

A significantly higher proportion of JN.1^§^ cases reported at least one risk factor for severe COVID-19 compared with BA.4/BA.5^§^ cases (Figure 4B). Additional details on the proportion of risk factors of JN.1^§^ cases compared with previously investigated cases are provided in Supplementary Table S3. Moreover, a higher proportion of previous SARS-CoV-2 infection was reported among JN.1^§^ cases than BA.4/BA.5^§^ cases (Table 2). The vaccination status of JN.1^§^ cases was significantly different to that of BA.4/BA.5^§^ cases. (Table 2).

### Comparison between JN.1^§^ and EG.5^§^ variants (RELAB data)

**Figure 5:**
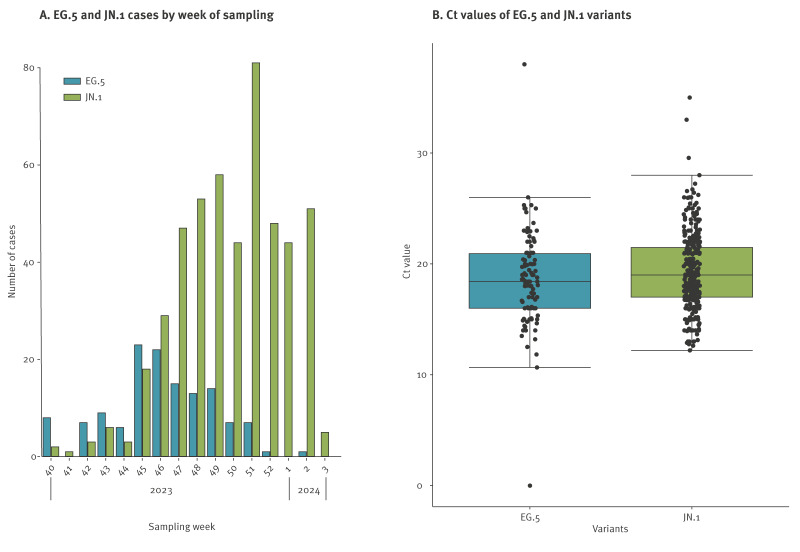
Number of cases by week of sampling (A) and distribution of Ct values (B) for variants EG.5^§^ (n = 133) and JN.1^§^ (n = 493), RELAB network, France, up to epidemiological week 3 of 2024

EG.5^§^ was the dominant SARS-CoV-2 variant before the emergence of JN.1^§^ and circulated in France between July and November 2023. From the end of September 2023 onward, JN.1^§^ detection increased, replacing EG.5^§^ as the main variant (Figure 5).

Ct: cycle threshold.

Between 2 October 2023 (week 40) and 18 January 2024 (week 3), sequencing data from RELAB database included 493 JN.1^§^ and 133 EG.5^§^ cases. No significant differences in demographic (sex, age) and clinical (fever, respiratory symptoms, vaccination status) characteristics were observed between JN.1^§^ and EG.5^§^ cases. Supplementary Table S4 provides the characteristics of individuals infected with these variants, in terms of sex, age, fever, respiratory symptoms, first respiratory symptoms, vaccination, time since last vaccination dose and Ct value. The median ages were 58 years and 55 years for JN.1^§^ and EG.5^§^ cases, respectively. The sex ratio was 0.6 females to males for both JN1^§^ and EG.5^§^ cases. Regarding vaccination status, 57% of both JN.1^§^ and EG.5^§^ cases declared being vaccinated against COVID-19, with a similar proportion of delay since last dose of vaccine between the two variants. No significant difference in Ct values between JN.1^§^ and EG.5^§^ was observed at diagnosis, as shown in Figure 5.

## Discussion

This study describes an early evaluation of the public health impact of the highly divergent JN.1 variant of SARS-CoV-2 in France in late 2023. In addition to combining data from routine surveillance systems, a novel surveillance network based on community laboratories, named RELAB, was mobilised. Combining data sources was challenging as they were all developed by different agencies and no linking by IDs was possible. Collaborations between SpF, NRC-VIR, sequencing laboratories from the EMERGEN consortium and other private and public partners from both public health and research were key to this investigation. Since the emergence of BA.2.86, results included in this paper have contributed to the various risk analyses on emerging SARS-CoV-2 variants produced by SpF and NRC-VIR, which were shared with the Ministry of Health and international partners to assess and discuss appropriate control measures.

The estimated doubling time of JN.1^§^ by the end of November varied between 8.6 and 26.4 days across French regions. The doubling time for three regions was highly uncertain; having complete data available for more time points likely would have improved estimates for these locations. The dynamics of JN.1^§^ spread in France was much less intense than that observed during the introduction in late 2021 of Omicron, for which the doubling time was estimated to be 2.2–2.7 days [12]. These differences are likely driven by different levels of population immunity, among other factors. For JN.1^§^, in vitro studies indicated higher rates of immune escape, but lower transmissibility compared with previous variants [13-15]. A study on COVID-19 vaccine effectiveness also suggested potential immune escape from XBB.1.5 vaccination and prior infection for JN.1 variant. [16,17].

As early as late August 2023, SpF initiated epidemiological investigations of BA.2.86^§^ cases. Of the first samples of BA.2.86^§^ detected by sequencing, we selected a convenience sample of cases to be interviewed. Although demographic characteristics were similar between all sequenced cases, interviewed cases and cases who were contacted but declined to be interviewed, we did not make comparisons because our convenience sample might not be generalisable to all COVID-19 cases. Over the study period, an increasing proportion of investigated BA.2.86^§^ cases corresponded to JN.1^§^. The demographic and vaccination data of JN.1^§^ cases were similar to other BA.2.86^§^ cases. The only difference noted was a higher frequency of anosmia among JN.1^§^ cases. These results are consistent with a study from Denmark which also found no difference in the symptoms of cases infected by JN.1^§^ compared with other BA.2.86^§^ sub-lineages [18].

The questionnaire used to investigate BA.2.86^§^ cases had already been used between April and June 2022 to investigate BA.4^§^ and BA.5^§^ cases [7]. When comparing the results of the two investigations, the different study periods is an important limitation. The geographic and age distribution were different between JN.1^§^ and BA.4/BA.5^§^ cases, with JN.1^§^ cases belonging to older age groups (> 54 years). This age shift might not be caused by a difference between the variants but might be linked to diagnosis biases: changes in testing behaviour could have led to an increasing proportion of symptomatic and at-risk people (including older individuals > 65 years) in the population which tested positive for SARS-CoV-2 with RT-PCR between 2022 and 2023. More JN.1^§^ cases reported a previous SARS-CoV-2 infection, which could be explained by the different study periods and longer exposure to SARS-CoV-2 for cases included during JN.1^§^ circulation. Recall bias might also have impacted reported vaccination status and previous infections (vaccination periods: August–November 2023 for JN.1^§^ vs April–May 2022 for BA.4/BA.5^§^). JN.1^§^ cases reported more frequent feverish feeling and less frequent cough or nausea compared with BA.4/BA.5^§^ cases.

JN.1^§^ cases were compared with those infected by the previously dominant variant EG.5^§^ using data from the RELAB network. RELAB data started in October 2023, which was later in the course of JN.1^§^ emergence. It was possible nonetheless to compare 103 EG.5^§^ cases with 343 JN.1^§^ cases diagnosed over the same period, and thus in the same population context. No significant differences between JN.1^§^ and EG.5^§^ cases were found in terms of clinical and virological characteristics [19,20]. Other studies comparing JN.1 vs non-JN.1 cases including EG.5 reported similar age, sex, comorbidities and reduced hospital admission rate with JN.1.

SIDEP and RELAB are new data sources that were developed during the COVID-19 pandemic. Based on the lessons learnt from implementing SIDEP, a new nation-wide laboratory-based information system is being designed, called LABOéSI. This new system will be able to automatically collect test results from laboratories in real time, starting with SARS-CoV-2 and later extending to other pathogens. When SIDEP collected only PCR results, RELAB data also included additional clinical and virological data (vaccination status, Ct values and presence of symptoms) and access to samples for genomic analysis or viral isolation. Although RELAB data are less detailed than case investigations, they are timelier (sent weekly by partner laboratories) and are less time-consuming to collect. At the time of this study, the RELAB network only included two private laboratory groups. Their distribution over the country covers all regions of mainland France but is more heterogeneous at the department level. In addition, they are not present in all overseas territories, which are critical locations for surveillance as their geographical location makes them vulnerable to different infectious disease threats. Further expansion of RELAB with the inclusion of other laboratory groups is ongoing to increase sample sizes and improve geographical coverage. This network will be invaluable for responding to future questions that might arise regarding SARS-CoV-2 and other respiratory viruses.

In the French context, public health microbiology falls under the mandate of the National Reference Center, which has an important coordinating role. For France, it is also of major importance to not overlook overseas territories. Surveillance networks that were set up during the COVID-19 pandemic, such as EMERGEN and RELAB, coupled with case investigation, have played a key role in SpF’s capacity to rapidly detect emerging variants and evaluate their potential public health impact in France. 

Using all available data sources, we showed that JN.1^§^ did not exhibit characteristics likely to increase its public health impact, especially in terms of severity and clinical presentation, compared with its parental lineage BA.2.86^§^ and previously circulating variants EG.5^§^ and BA.4/BA.5^§^. Since this analysis was performed, more in vitro studies using viruses from clinical isolates became available and showed no major differences in term of transmissibility and immune escape between BA.2.86^§^ and recent Omicron sub-lineages, such as XBB.1.5^§^, XBB.1.16^§^ and EG.5^§^ [21]. A German study based on wastewater and hospital data concluded that JN.1^§^ emergence had an impact on case incidence but not on case mortality [22,23]. In its risk assessments from December 2023 and February 2024, WHO described JN.1^§^ as posing a ‘low’ risk to global public health [4,24]. Thus, no additional control measures were implemented in France following the emergence of JN.1^§^.

## Conclusions

Although JN.1^§^ emergence was not associated with an increased public health impact compared with previously circulating variants, new SARS-CoV-2 variants continue to emerge. For this reason, WHO encourages countries to maintain a genomic surveillance system. Future challenges will include the use of what was learnt and set-up during the COVID-19 pandemic to build efficient and resilient surveillance systems and be able to respond to future crises. An improved system would require robust laboratory infrastructures for testing, continuous genomic sequencing capabilities, and advanced bioinformatics resources for data analysis. Regulatory issues, such as data privacy and ethical considerations, must be addressed to facilitate linking datasets and implement effective surveillance processes before the next crisis. Data from community and hospital settings should be integrated into a unique national database, ideally shared across public health actors, to enable comprehensive and timely analyses. In addition to infrastructures and capacities, the response to COVID-19 also included collaborations, such as the EMERGEN consortium and the RELAB community laboratories network. Maintaining such collaborations in between crises will provide a more robust foundation to respond to future health emergencies.

## Members of the RELAB Study Group

Arnaud François (Bioesterel, Biogroup PACA); Alexandre Vignola (Oriade Noviale, Biogroup AURA); Vincent Garcia (Alphabio, Biogroup PACA); Alexandra Jacques (Biogroup Lorraine, Grand Est); Jonas Amzalag (Biolam, Biogroup IDF); Nadège Gourgouillon (CAB Biogroup, Grand Est); Remi Labetoulle (Laborizon, Biogroup Nouvelle Aquitaine); Frederique Roumanet (Unilians, Biogroup AURA); Arthur Denoel (Laborizon, Biogroup Bretagne); Hilel Mehamha (CBM25, Biogroup Bourgogne Franche-Comté); Thierry Guffond (Diagnovie, Biogroup Hauts de France); Magali HYPOLITE (2A2B, Biogroup Corse); Yanis Chaib (Biolam, Biogroup IDF); Timothée Goetschy (Biogroup national, équipe Data); Elodie Ostermann (Biogroup national, équipe Data); Anne Holstein (Laborizon Biogroup Centre Val de Loire); Vincent Vieillefond (BPO-Bioépine Biogroup IDF); Jean Marc Giannoli (Biogroup national); Julienne de Pontcharra (Bioesterel, Biogroup PACA); Jean Francois Comes (Laborizon, Biogroup Bretagne); Justine Gasnot (Bioesterel, Biogroup PACA); Theo Corbet (Unilians, Biogroup AURA); Laurent Kbaier (Bioesterel, Biogroup PACA); Emmanuel Chanard (Cerballiance, Auvergne Rhône Alpes); Arcadie Gioud (Cerba Xpert, Auvergne Rhône Alpes); Stéphanie Arsene (Cerballiance, Normandie Bocage); Maxime Sansot (Cerballiance, Pays de Loire); Anne-Lise Gautier (Cerballiance, Portes de Bretagne); Kariach Goldar (Cerballiance, Martinique); Mahery Ramiandrisoa (Cerballiance, Réunion); Aristide Nzeumi (Cerballiance, Réunion); Pauline Jestin (Cerballiance, Charentes); Gilles Abs (Cerballiance, Centre Val-de-Loire); Guillemette Wandler (Cerballiance, Centre Val-de-Loire); Anne-Claire Strzelecki (Cerballiance, Occitanie); Sarah Cerdan (Cerballiance, Occitanie); Edouard Delaunay (Cerballiance, Alpes Durance); Sandrine Barrieu-Moussat (Cerballiance, Côte d'azur); Laurence Prots (Cerballiance, Côte d'azur); Edouard Delaunay (Cerballiance, Provence); Johanna Roux (Cerballiance, Ile-de-France Est); Yasmina De Saint Salvy (Cerballiance, Ile-de-France Est); Agnes Durand (Cerballiance, Ile-de-France Est); Aude Lesenne (Cerballiance, Ile-de-France Sud); Kader Merah (Cerballiance, Ile-de-France Sud); Erwan Le Naour (Cerballiance, Aquitaine Nord); David Robert (Cerballiance, Aquitaine Nord); Sophie Zaffreya (Cerballiance, Aquitaine Nord); Jean-Philippe Galhaud (Cerballiance, Aquitaine Sud); Claire Felloni (Cerballiance, Artois); Dominique Dyda (Cerballiance, Haut-de-France); Aurelie Dupuis (Cerballiance, Bretagne); Gwenole Prigent (Cerballiance, Bretagne); Stephanie Arsene (Cerballiance, Normandie Ouest); Antoine Prigent (Cerballiance, Normandie Ouest); Natacha Tatoyan (Cerballiance, Nouvelle Calédonie); Benoit Visseaux (Laboratoire Cerba, Pole Infectiologie); Bénédicte Roquebert (Laboratoire Cerba, Pole Infectiologie); Stéphanie Haim-Boukobza (Cerba Healthcare); Odile Rousselet (Cerba Healthcare) and Michel Sala (Cerba Healthcare).

## Supplementary Data

938000 Supplement
