# Supplementary material for: Monitoring SARS-CoV-2 variants with complementary surveillance systems: risk evaluation of the Omicron JN.1 variant in France, August 2023 to January 2024
Source: Euro Surveill. 2025 Jan 9;30(1):2400293. doi: 10.2807/1560-7917.ES.2025.30.1.2400293 (PMC11719805; doi:10.2807/1560-7917.ES.2025.30.1.2400293)
Supplement: Supplement [file 24-00293_DACRUZ_Supplement.pdf]

Supplementary material

This supplementary material is hosted by Eurosurveillance as supporting information alongside the article **Monitoring SARS-CoV-2 variants with complementary surveillance systems: risk evaluation of the Omicron JN.1 variant in France, August 2023 to January 2024** on behalf of the authors who remain responsible for the accuracy and appropriateness of the content. The same standards for ethics, copyright, attributions and permissions as for the article apply. Eurosurveillance is not responsible for the maintenance of any links or email addresses provided therein.

**S1.** Characteristics of BA.2.86[§] cases identified in France with a sampling date between 21 August 2023 and 05 November 2023 (n = 638, data from EMERGEN-DB) depending whether they were investigated or not.

| Characteristics[1] | **Investigated BA.2.86[§] case (n = 147)** | | **Detected BA.2.86[§] case not investigated (n = 491)** | |
|---|---|---|---|---|
| **Variant** | | | | |
| JN.1[§] | 74 | (50.3%) | 351 | (71.5%) |
| Other BA.2.86[§] | 73 | (49.7%) | 140 | (28.5%) |
| **Detailed Pango lineage** | | | | |
| BA.2.86 | 0 | (0%) | 4 | (0.8%) |
| BA.2.86.1 | 54 | (36.7%) | 84 | (17.1%) |
| BA.2.86.2 | 2 | (1.4%) | 3 | (0.6%) |
| BA.2.86.3 | 0 | (0%) | 1 | (0.2%) |
| JN.1 | 74 | (50.3%) | 351 | (71.5%) |
| JN.2 | 6 | (4.1%) | 24 | (4.9%) |
| JN.3 | 7 | (4.8%) | 13 | (2.6%) |
| JQ.1 | 4 | (2.7%) | 11 | (2.2%) |
| **Sex** ($n_1$=147, $n_2$=490) | | | | |
| Female | 90 | (61.2%) | 273 | (55.8%) |
| Male | 57 | (38.8%) | 216 | (44.1%) |
| **Age** ($n_1$=146, $n_2$=489) | | | | |
| Median (IQR) | 58.5 | (42.3 - 71.8) | 64.0 | (48.0 - 82.0) |
| Range (min – max) | | 0.0 - 100.0 | | 0.0 - 122.0 |
| **Region of residence** | | | | |
| Auvergne-Rhône-Alpes | 12 | (8.2%) | 64 | (13.0%) |
| Bourgogne-Franche-Comté | 15 | (10.2%) | 23 | (4.7%) |
| Bretagne | 4 | (2.7%) | 7 | (1.4%) |
| Centre-Val de Loire | 16 | (10.9%) | 36 | (7.3%) |
| Corsica | 3 | (2.0%) | 5 | (1.0%) |
| Grand Est | 12 | (8.2%) | 63 | (12.8%) |
| Hauts-de-France | 13 | (8.8%) | 39 | (7.9%) |
| Île-de-France | 37 | (25.2%) | 133 | (27.1%) |
| La Réunion | 12 | (8.2%) | 8 | (1.6%) |
| Mayotte | 0 | (0%) | 11 | (2.2%) |
| Normandie | 1 | (0.7%) | 10 | (2.0%) |
| Nouvelle-Aquitaine | 2 | (1.4%) | 31 | (6.3%) |
| Occitanie | 8 | (5.4%) | 39 | (7.9%) |
| Pays de la Loire | 2 | (1.4%) | 12 | (2.4%) |
| Provence-Alpes-Côte d'Azur | 10 | (6.8%) | 10 | (2.0%) |

[1] For each category, percentages have been calculated for the number of cases with available data

Supplementary material

**S2.** Proportion of symptoms of JN.1§ cases compared to previously investigated BA.2.86§ cases and JN.1§ compared to BA.4/BA.5§.

| | **JN.1§** (n = 70)[1] | | **Other BA.2.86§** (n = 71)[1] | | p-value[2] | **BA.4/BA.5§** (n = 277)[1] | | p-value[2] |
|---|---|---|---|---|---|---|---|---|
| Asthenia/Fatigue | 47 | (67.1%) | 48 | (67.6%) | >0.9 | 204 | (73.6%) | 0.09 |
| **Cough** | 33 | (47.1%) | 44 | (62.0%) | 0.08 | **162** | **(58.5%)** | **0.03** |
| Fever | 40 | (57.1%) | 30 | (42.3%) | 0.08 | 156 | (56.3%) | 0.7 |
| Headache | 35 | (50.0%) | 30 | (42.3%) | 0.4 | 142 | (51.3%) | 0.5 |
| Rhinorrhoea | 35 | (50.0%) | 28 | (39.4%) | 0.2 | 135 | (48.7%) | 0.8 |
| Myalgia | 28 | (40.0%) | 30 | (42.3%) | 0.8 | 113 | (40.8%) | 0.6 |
| Sore throat | 26 | (37.1%) | 24 | (33.8%) | 0.7 | 110 | (39.7%) | 0.5 |
| **Feverish feeling** | 23 | (32.9%) | 21 | (29.6%) | 0.7 | **52** | **(18.8%)** | **0.02** |
| Shortness of breath | 10 | (14.3%) | 16 | (22.5%) | 0.2 | 44 | (15.9%) | 0.6 |
| **Anosmia** | **15** | **(21.4%)** | **6** | **(8.5%)** | **0.03** | 44 | (15.9%) | 0.4 |
| Diarrhoea | 7 | (10.0%) | 11 | (15.5%) | 0.3 | 43 | (15.5%) | 0.2 |
| Ageusia | 9 | (12.9%) | 7 | (9.9%) | 0.6 | 49 | (17.7%) | 0.3 |
| **Nausea/Vomiting** | 4 | (5.7%) | 4 | (5.6%) | >0.9 | **50** | **(18.1%)** | **0.007** |
| Dyspnoea | 3 | (4.3%) | 5 | (7.0%) | 0.7 | 21 | (7.6%) | 0.3 |
| Abnormal pulmonary auscultation (APA) | 0 | (0%) | 1 | (1.4%) | >0.9 | 1 | (0.4%) | >0.9 |
| Acute respiratory distress syndrome (ARDS) | 0 | (0%) | 0 | (0%) | - | 1 | (0.4%) | >0.9 |

[1] For each category, percentages have been calculated for the number of cases with available data

[2] Chi-square test for categorical variables (Fisher's exact test for small theoretical numbers)

n1=number of JN.1§ cases. n2=number of other BA.2.86§ cases. n3=number of BA.4/BA.5§ cases

Bold indicates statistical significance at the 0.05 level.

**S3.** Proportion of risk factors of JN.1§ cases compared to previously investigated BA.2.86§ cases and JN.1§ compared to BA.4/BA.5§.

| | **JN.1§** (n = 33)[1] | | **Other BA.2.86§** (n = 35)[1] | | p-valeur[2] | **BA.4/BA.5§** (n=78)[1] | | p-valeur[2] |
|---|---|---|---|---|---|---|---|---|
| Hypertension | 8 | (24.2%) | 15 | (42.9%) | 0.1 | 28 | (35.9%) | 0.2 |
| Diabetes (type 1 or 2) | 9 | (27.3%) | 10 | (28.6%) | >0.9 | 20 | (25.6%) | 0.9 |
| Cardiac pathology | 4 | (12.1%) | 9 | (25.7%) | 0.2 | 17 | (21.8%) | 0.3 |
| Chronic respiratory pathology | 3 | (9.1%) | 2 | (5.7%) | 0.7 | 11 | (14.1%) | 0.5 |
| Obesity (BMI > 30) | 2 | (6.1%) | 4 | (11.4%) | 0.7 | 11 | (14.1%) | 0.3 |
| Kidney failure | 3 | (9.1%) | 5 | (14.3%) | 0.7 | 4 | (5.1%) | 0.4 |
| Progressive cancer under treatment | 3 | (9.1%) | 1 | (2.9%) | 0.3 | 2 | (2.6%) | 0.2 |
| Asthma | 3 | (9.1%) | 0 | (0%) | 0.7 | 1 | (1.3%) | 0.08 |
| Pregnancy | 2 | (6.1%) | 1 | (2.9%) | 0.1 | 1 | (1.3%) | 0.2 |
| Neuromuscular pathology | 1 | (3.0%) | 1 | (2.9%) | >0.9 | 3 | (3.8%) | >0.9 |
| Immunosuppression | 1 | (3.0%) | 0 | (0%) | 0.5 | 3 | (3.8%) | >0.9 |
| Liver pathology | 0 | (0%) | 2 | (5.7%) | 0.5 | 0 | (0%) | - |

[1] For each category, percentages have been calculated for the number of cases with available data

[2] Chi-square test for categorical variables (Fisher's test for small theoretical numbers)

n1=number of JN.1§ cases. n2=number of others BA.2.86§ cases. n3=number of BA.4/BA.5§ cases

Supplementary material

**S4.** Characteristics of infection by JN.1§ and EG.5§ from the RELAB data, sampling date from 02/10/2023 to 18/01/2024, mainland France.

| | **JN.1§ ($n_1$ = 493)** | | **EG.5§ ($n_2$ = 133)** | | **p-value[1]** |
|---|---|---|---|---|---|
| **Date of sampling** | | | | | |
| Range | 02-10-2023 to 18-01-2024 | | 02-10-2023 to 08-01-2024 | | |
| **Sex** | | | | | 0.86[3] |
| Female | 296 | (60%) | 81 | (61%) | |
| Male | 197 | (40%) | 52 | (39%) | |
| **Age** | | | | | |
| Median (IQR) | 58 | (45 - 72) | 55 | (41- 71) | 0.41[2] |
| **Fever** | | | | | 0.14[3] |
| Yes | 199 | (40%) | 60 | (45%) | |
| No | 151 | (31%) | 29 | (22%) | |
| Not specified | 143 | (29%) | 44 | (33%) | |
| **Respiratory symptoms** | | | | | 0.98[3] |
| Yes | 246 | (50%) | 67 | (50%) | |
| No | 113 | (23%) | 31 | (23%) | |
| Not specified | 134 | (27%) | 35 | (26%) | |
| **First respiratory symptoms** (From the RT-PCR) | | | | | 0.71[4] |
| less than 24 hours | 58 | (12%) | 13 | (9.8%) | |
| 2 to 5 days | 135 | (27%) | 33 | (25%) | |
| 6 to 10 days | 13 | (2.6%) | 2 | (1.5%) | |
| Not specified | 287 | (58%) | 85 | (64%) | |
| **Vaccination** | | | | | 0.52[3] |
| Yes | 280 | (57%) | 76 | (57%) | |
| No | 40 | (8.1%) | 7 | (5.3%) | |
| Not specified | 173 | (35%) | 50 | (38%) | |
| **Time since last vaccination dose** | | | | | 0.33[4] |
| <15 days | 4 | (0.8%) | 0 | (0%) | |
| 15 days-3 months | 14 | (2.8%) | 2 | (1.5%) | |
| 3 to 6 months | 9 | (1.8%) | 0 | (0%) | |
| >6 months | 191 | (39%) | 50 | (38%) | |
| Not vaccinated | 40 | (8.1%) | 7 | (5.3%) | |
| Not specified | 173 | (35%) | 50 | (38%) | |
| Vaccinated but time unknown | 62 | (13%) | 24 | (18%) | |
| **Ct value (**technique Eurobio) ($n_1$=337, $n_2$=102) | | | | | 0.18[2] |
| Median (IQR) | 19.0 | (17.0 - 21.5) | 18.4 | (16.0 - 20.9) | |

[1] *p<0.05; **p<0.01; ***p<0.001

[2] Wilcoxon rank sum test

[3] Pearson's Chi-squared test

[4] Fisher's exact test

n1=number of JN.1§ cases, n2=number of EG.5§ cases

Supplementary material

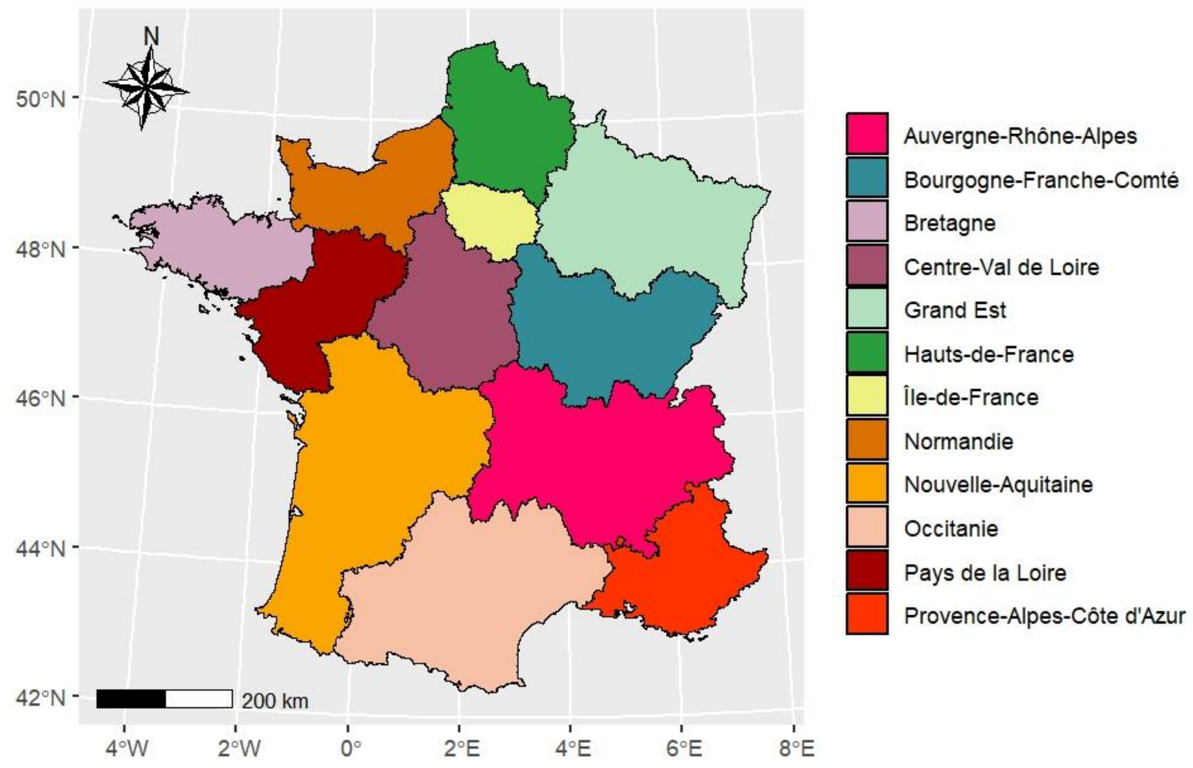


**Figure S1**. Map of the 12 French regions included in mainland France (excluding Corsica, an island region located in the Mediterranean Sea). The map was made with shapefiles from the ADMIN EXPRESS data product, which is produced by Institut National de l'Information Géographique et Forestière. The shapefiles were last updated on July 30, 2024, and the only modification to them was the removal of Corsica. The shapefiles are available under an open license 2.0 on the open platform for French public data (https://www.data.gouv.fr/fr/datasets/admin-express/#/resources).

Supplementary material

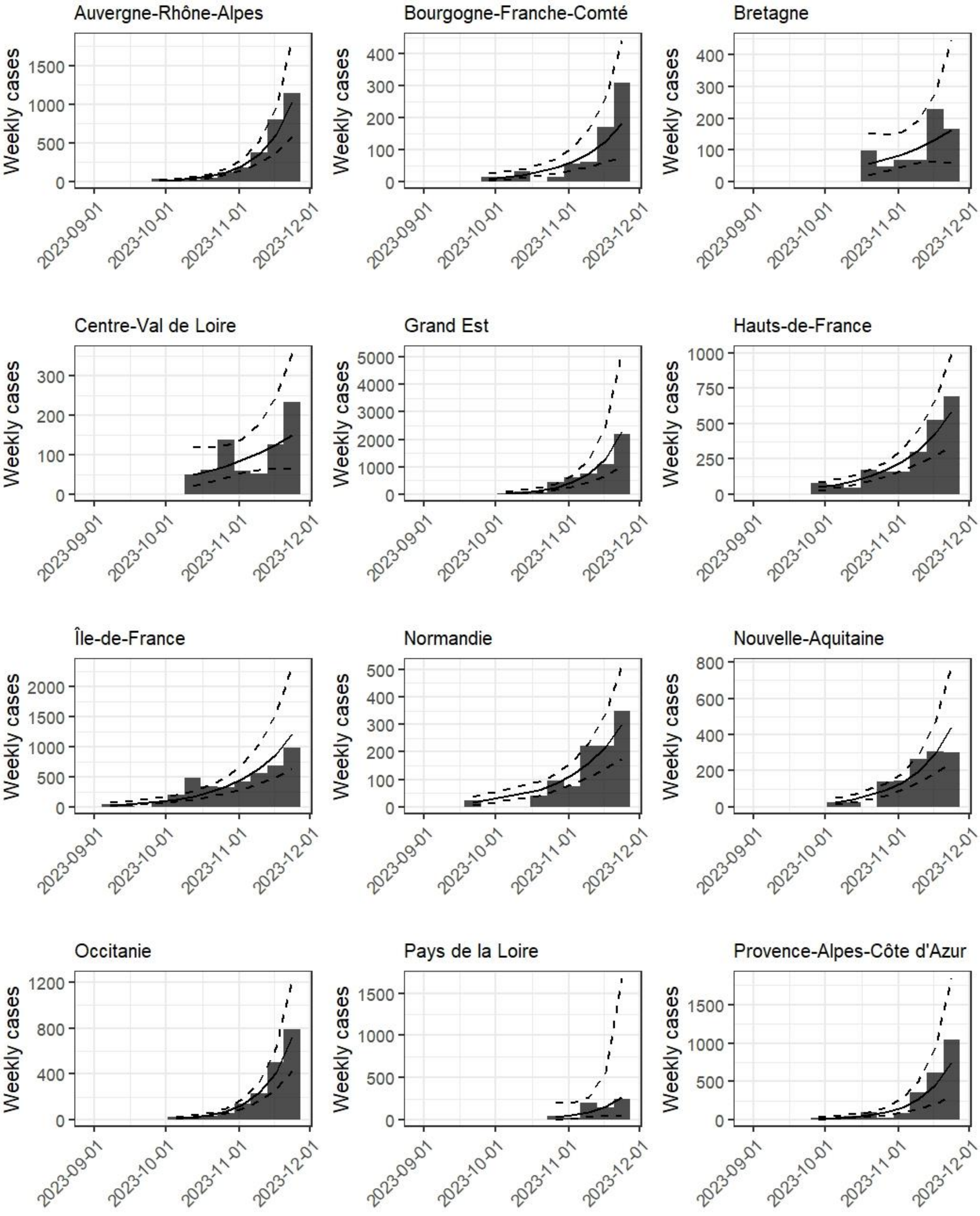


**Figure S2**. Incidence predictions and 95% confidence intervals from log-linear models for COVID-19 cases due to the JN.1 variant across the 12 regions of mainland France, September 4 – November 20, 2023 (weeks 36–47). The estimates for Bretagne, Centre-Val de Loire, and Pays de la Loire are highly uncertain. Note that the y axes are different in each plot.

Supplementary material

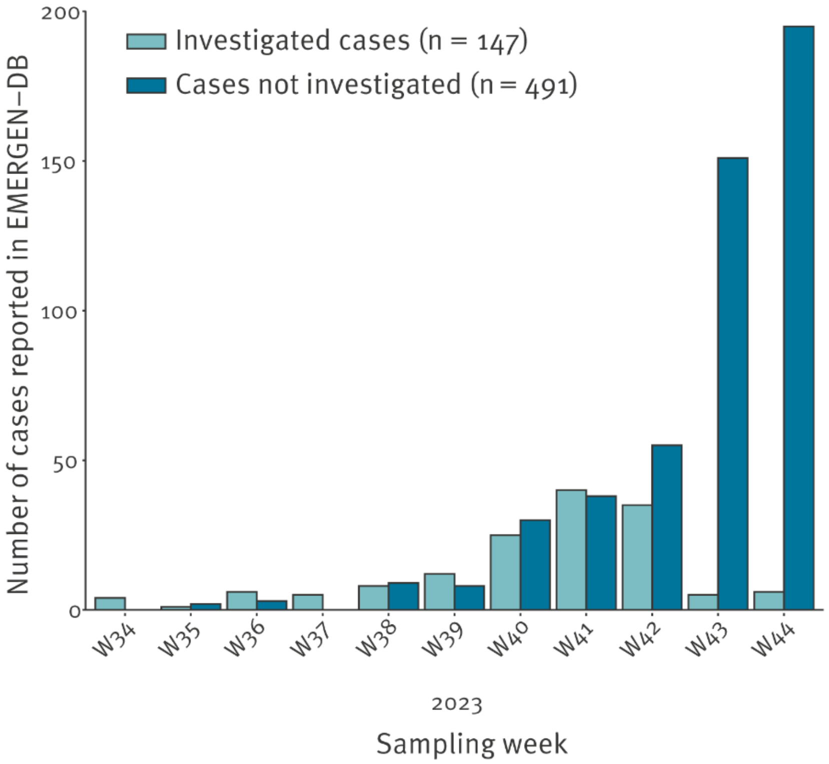


**Figure S3**. Time distribution of BA.2.86 sequences and BA.2.86 cases that were investigated and uploaded to EMERGEN-DB, by sampling week.
